# Self-Efficacy as a Common Pathway: How Non-Cognitive Individual Differences Shape Academic Procrastination

**DOI:** 10.3390/jintelligence14080188

**Published:** 2026-08-17

**Authors:** Şule Betül Tosuntaş

**Affiliations:** Department of Educational Sciences, Bursa Uludağ University, Bursa 16059, Turkey; sbtosuntas@uludag.edu.tr

**Keywords:** academic procrastination, general self-efficacy, automatic thoughts, perfectionism, locus of control, latent profile analysis, self-regulation

## Abstract

Academic procrastination is a widespread self-regulatory failure among university students and a costly one for both academic achievement and psychological well-being. In this study, procrastination was examined within the framework of non-cognitive individual differences. The model tested here posits that perfectionism and locus of control affect procrastination through negative automatic thoughts and general self-efficacy. These variable-centered analyses were then complemented with a person-centered approach in order to capture distinct risk profiles. Cross-sectional data were collected from 1003 Turkish university students (71.5% women; *M*_age_ = 21.93 years) and analyzed with structural equation modeling, latent profile analysis (LPA), and random forest regression. The structural model accounted for 62% of variance in procrastination (CFI = 0.958, RMSEA = 0.074). Based on these results, general self-efficacy was identified as a common pathway through which distal non-cognitive influences converge on procrastination. The direct effect of automatic thoughts was non-significant, whereas the indirect effect through self-efficacy was strong (indirect effect = 0.51, 95% CI [0.44, 0.58]). LPA identified three profiles: adaptive, average, and vulnerable; the vulnerable profile showed markedly higher procrastination than the adaptive profile (d = 1.21). Random forest analysis indicated no meaningful nonlinearity, with self-efficacy as the dominant predictor. These findings suggest that interventions may benefit from prioritizing general self-efficacy beliefs.

## 1. Introduction

Academic procrastination, defined as the voluntary delay of academic tasks despite awareness of the negative consequences of doing so, is among the most pervasive and consequential self-regulatory difficulties in higher education (Steel, 2007). Meta-analytic estimates indicate that approximately 50% of university students procrastinate chronically, with roughly 25% reporting it as a severe and ongoing problem (Steel, 2007). The behavioral and psychological costs of chronic procrastination are well established: it is associated with lower academic performance, higher dropout rates, elevated stress and anxiety, depressive symptomatology, and diminished life satisfaction (Solomon & Rothblum, 1984; Steel, 2007; Van Eerde, 2003). Given the scope of its impact, understanding which individual differences predict procrastination and the mechanisms through which they operate remains both theoretically important and practically urgent.

Procrastination as Self-Regulatory Failure

Contemporary theories view procrastination as a failure of self-regulation, since the individual is unable to translate intentions into timely action (Baumeister & Heatherton, 1996; Steel, 2007). Steel and König (2006), in their Temporal Motivation Theory, explain this through four competing forces: task utility, task aversiveness, temporal discounting, and expectancy. When expectancy (the individual’s belief that effort will lead to success) is low, even useful tasks are postponed and more immediately rewarding alternatives are pursued instead. In this sense, procrastination is explained not as a time-management problem but as a systematic motivational and cognitive failure rooted in individual differences (Steel, 2007; Steel & Klingsieck, 2016; Wolters, 2003). Identifying the factors that predict procrastination, and the processes that mediate their effects, is therefore important for the development of intervention programs.

Non-Cognitive Individual Differences in Academic Functioning

A substantial body of research now demonstrates that academic functioning is shaped by a broad constellation of non-cognitive individual differences, extending well beyond cognitive ability (Duckworth & Seligman, 2005; Zimmerman, 2000). In a seminal investigation, Duckworth and Seligman (2005) showed that self-discipline predicted academic outcomes more strongly than IQ among adolescents, catalyzing broader interest in the non-cognitive architecture of academic success and failure. Constructs such as self-efficacy beliefs, attributional style, conscientiousness, and self-regulation strategies have each been shown to account for unique variance in academic outcomes above and beyond measured cognitive ability (Zimmerman, 2000). Within this framework, academic procrastination can be positioned as a prototypical non-cognitive deficit: a systematic self-regulatory failure that undermines academic functioning regardless of the individual’s intellectual capacity. Studying the non-cognitive antecedents of procrastination thus speaks to a central concern of intelligence and individual-differences research: namely, which stable psychological characteristics, other than cognitive ability, shape academic behavior and achievement? Large-scale syntheses indicate that such non-cognitive constructs predict academic performance incrementally over measured cognitive ability (Poropat, 2009; Richardson et al., 2012), which makes clarifying the mechanisms through which they operate a question of direct relevance to individual-differences and intelligence research.

Perfectionism and Procrastination

Perfectionism is a multidimensional personality disposition characterized by the tendency to set excessively high personal standards and to evaluate one’s own performance with harsh self-criticism (Frost et al., 1990). Frost and colleagues identified six components of perfectionism (concern over mistakes, doubts about actions, personal standards, parental criticism, parental expectations, and organization), of which concern over mistakes and doubts about actions are most consistently linked to maladaptive outcomes. The theoretical link between perfectionism and procrastination is intuitive: individuals who fear making mistakes may defer task initiation as a self-protective strategy, thereby avoiding the negative affect that accompanies perceived imperfection (Ferrari, 1992; Schouwenburg, 1995). An important implication of this account is that perfectionism promotes avoidance not because the individual lacks motivation but because the anticipated negative consequences of potential failure (evaluation anxiety, self-criticism, shame) render task engagement aversive.

Despite this theoretical clarity, the empirical relationship between perfectionism and procrastination has proven surprisingly inconsistent. Van Eerde’s (2003) meta-analysis reported only modest associations across studies, and the direct correlation between perfectionism and procrastination is frequently small or statistically unreliable. This inconsistency suggests that perfectionism’s influence on procrastination may be primarily indirect, operating through cognitive and motivational intermediaries rather than through a straightforward trait–behavior link. The present study addresses this possibility directly by situating perfectionism as a distal antecedent whose effects on procrastination are transmitted through more proximal cognitive and motivational mechanisms. Because the present model positions perfectionism as a distal dispositional antecedent rather than as a differentiated set of mechanisms, total FMPS scores were used; accordingly, the present findings speak to perfectionism as a global disposition and do not permit inferences about specific adaptive or maladaptive dimensions.

Locus of Control and Procrastination

Locus of control, originally conceptualized by Rotter (1966), refers to generalized expectancies regarding whether outcomes in one’s life are determined by one’s own behavior and attributes (internal locus) or by external forces such as luck, chance, or powerful others (external locus). Individuals with an external locus of control perceive the contingency between behavior and outcomes as low, undermining the motivational basis for sustained self-regulatory effort. When outcomes are seen as independent of one’s actions, initiating or persisting with aversive tasks carries little perceived instrumental value, a state highly conducive to procrastination (Rotter, 1966; Steel & König, 2006). Empirical research confirms a positive association between external locus of control and procrastination, though the effect sizes are generally modest (Van Eerde, 2003), suggesting that locus of control functions as a distal dispositional antecedent whose influence on procrastination is mediated by more proximal cognitive and motivational processes.

Negative Automatic Thoughts and Procrastination

Negative automatic thoughts, the rapid, involuntary, negatively valenced self-referential cognitions that characterize anxious and depressive states, represent a more proximal cognitive mechanism implicated in self-regulatory failure (Beck et al., 1979; Hollon & Kendall, 1980). Grounded in Beck’s cognitive model, automatic thoughts are understood as schema-driven appraisals that color the individual’s perception of their own competence, the likelihood of successful task completion, and the anticipated consequences of failure (Beck et al., 1979). Thought patterns such as “I am unable to do this” or “No matter what I do, it will not be good enough” directly undermine the expectancy component of motivated behavior, increasing the perceived futility of effort and the subjective aversiveness of task engagement. In the context of procrastination, negative automatic thoughts erode the motivational conditions necessary for task initiation and persistence, making delay an increasingly attractive option for short-term mood repair (Sirois & Pychyl, 2013).

Importantly, negative automatic thoughts are not independent of more distal individual differences. Perfectionistic concerns, particularly those centered on mistakes and performance doubts, are well-documented antecedents of negative automatic thinking (Frost et al., 1990). Similarly, external locus of control, by promoting the perception that one’s outcomes are determined by forces outside personal control, may amplify negative self-referential cognitions in achievement contexts. Automatic thoughts thus plausibly function as a cognitive link between distal personality dispositions and more proximal self-regulatory processes.

General Self-Efficacy as a Common Pathway

Within Bandura’s (1997) social cognitive theory, self-efficacy (an individual’s belief in their capacity to organize and execute the courses of action required to produce specific attainments) is a central determinant of motivation, goal-setting, effort, persistence, and behavioral regulation. Self-efficacy beliefs influence the challenges individuals take on, the amount of effort they invest, how long they persevere in the face of obstacles, and how they respond to failure. Low self-efficacy, specifically, promotes behavioral avoidance and task delay: when individuals doubt their capacity to successfully execute required behaviors, the motivational value of effort diminishes and task avoidance becomes more likely (Bandura, 1997; Klassen et al., 2008). Klassen et al. (2008) provided direct evidence that self-efficacy to self-regulate is a stronger predictor of procrastination among undergraduates than several other motivational constructs. More recent work has continued to place self-efficacy in a mediating position: academic self-efficacy has been shown to mediate the association between procrastination and life satisfaction (Demir & Kuşcu Karatepe, 2025) and between negative academic emotions and delay (B. Chen et al., 2024), while general self-efficacy has been found to mediate links between dispositional resources and outcomes in domains outside the classroom (Tan et al., 2025). What remains unclear is whether these separate mediating roles reflect a single convergent mechanism.

The present study employs a measure of general self-efficacy (Sherer et al., 1982), that is, domain-unspecific beliefs about one’s overall capacity for effective action, rather than a measure of self-efficacy tied to specific academic tasks. This distinction is theoretically meaningful. General self-efficacy reflects a relatively stable, trait-like motivational orientation that should be sensitive to the accumulated influence of diverse upstream individual differences, including personality dispositions (perfectionism), generalized control beliefs (locus of control), and persistent cognitive tendencies (automatic thoughts). As a domain-general construct, it is positioned to integrate these heterogeneous upstream effects and transmit them to behavior in a consistent manner across task domains. Although procrastination is an academic behavior, the antecedents examined here (perfectionism, generalized control beliefs, and habitual negative cognition) are themselves domain-general dispositions; a domain-general efficacy construct is therefore the theoretically appropriate mediator, since a task-specific measure would capture only the academic expression of a broader motivational orientation.

The central thesis of the present study is that general self-efficacy may function as a common pathway through which the effects of distal non-cognitive individual differences converge on procrastination (Bandura, 1997). Within this mediation model, perfectionism and locus of control are expected to operate as distal antecedents, shaping negative automatic thinking, which in turn erodes general self-efficacy beliefs, which in turn predicts procrastination. The direct effects of more distal variables on procrastination, bypassing self-efficacy, are expected to be substantially attenuated or non-significant when self-efficacy is included as a mediator.

### The Present Study

Despite extensive studies on each of the constructs reviewed above, to our knowledge these four constructs have not previously been examined together within a single theoretically derived mediation model in which general self-efficacy is positioned as the proximal mechanism, nor has the convergence of their effects on a common pathway been tested directly. Moreover, existing research has been predominantly variable-centered, describing average relationships among constructs within a population. The four constructs examined here were selected because each occupies a distinct position in the proposed sequence rather than because they are the strongest known correlates of procrastination. Conscientiousness and impulsivity show larger meta-analytic associations with delay (Steel, 2007) but are broad temperamental dispositions that offer little purchase on the cognitive and motivational steps through which they operate; time-management skill, similarly, describes the behavior to be explained more than its antecedents. Perfectionism and locus of control were chosen as distal dispositions whose effects on delay are theoretically indirect and empirically inconsistent and automatic thoughts and general self-efficacy as the cognitive and motivational processes most plausibly linking them to behavior. The aim was thus explanatory rather than predictive: to specify a mechanism, not to maximize variance accounted for. Whether these relationships give rise to identifiable distinct subgroups of individuals with different risk configurations, a question with direct implications for targeted intervention, has not been examined in the context of this particular constellation of non-cognitive individual differences. The contribution is therefore twofold: the model specifies an ordering among predictors that are usually examined in isolation, and it tests that ordering at both the variable and the person level, which allows the question of whether an average pathway corresponds to identifiable groups of students to be addressed directly rather than assumed.

The present study addresses these gaps by combining three analytic approaches, each answering a distinct question about the same model rather than providing parallel tests of a single one. Variable-centered structural equation modeling addresses the question of how these constructs are related on average and provides the test of the proposed mediation pathways (H1–H3). Person-centered latent profile analysis (Spurk et al., 2020) addresses a question that variable-centered methods cannot: whether the average pathways correspond to identifiable configurations of individuals, or whether they instead describe a population in which no distinct risk groups exist (H4). This distinction matters for intervention, since pathway-based and profile-based targeting imply different allocation strategies (Spurk et al., 2020). Profile-based approaches have recently been applied to related academic constructs, including academic stress and self-efficacy (Zhao, 2024), but not to the present constellation of predictors. Finally, because both approaches assume that the modeled associations are adequately captured by linear functions, a cross-validated random forest regression was used as a specification check: machine learning methods impose no functional form and can therefore detect nonlinearity or higher-order interactions that a linear structural model would miss (Breiman, 2001; Yarkoni & Westfall, 2017). The three approaches thus stand in a defined relationship to one another, with the structural model as the primary test, the profile analysis as a person-level counterpart, and the random forest as a check on the assumptions underlying both. The following hypotheses were tested:

**Hypothesis** **1** **(H1).** *Negative automatic thoughts, perfectionism, and external locus of control will significantly and negatively predict general self-efficacy.*

**Hypothesis** **2** **(H2).** *General self-efficacy will significantly and negatively predict procrastination.*

**Hypothesis** **3** **(H3).** *The effects of perfectionism, locus of control, and automatic thoughts on procrastination will be significantly mediated by general self-efficacy.*

**Hypothesis** **4** **(H4).** *Latent profile analysis will reveal qualitatively distinguishable risk profiles that differ significantly in procrastination level.*

## 2. Materials and Methods

### 2.1. Participants

Participants were undergraduate students enrolled at a large state university in Türkiye. Data were collected in 2026 via an online survey platform. Recruitment was conducted through course announcements and institutional mailing lists, and participation was entirely voluntary. Eligibility was limited to actively enrolled undergraduate students who provided informed consent prior to participation; no incentives, course credit, or other compensation were offered. An initial sample of 1090 students participated. After application of the data-quality criteria described below, all analyses were conducted on a final sample of 1003 participants. Of these, 717 (71.5%) were women and 283 (28.2%) were men; gender information was missing for 3 participants (0.3%). The mean age was 21.93 years (SD = 4.52; range = 17–48). Participants were distributed across the years of study as follows: first year, 391; second year, 38; third year, 197; fourth year, 335; and year of study was not reported by 42 participants (4.2%). Participants were assured of anonymity and confidentiality throughout, and no personally identifying information was collected.

### 2.2. Measures

Academic Procrastination Scale: Academic procrastination was measured using the 14-item unidimensional scale developed by Tuckman (1991) and adapted into Turkish by Özer et al. (2013). Items are rated on a 4-point Likert scale (1 = strongly disagree, 4 = strongly agree), with four items reverse-scored. A sample item is “I needlessly delay finishing jobs, even when they’re important.” Higher scores indicate greater academic procrastination. Cronbach’s alpha in the present sample was 0.91.

Automatic Thoughts Questionnaire: Negative automatic thoughts were assessed using the 30-item Automatic Thoughts Questionnaire (ATQ; Hollon & Kendall, 1980), which measures how frequently each negative self-referential cognition occurred over the past week on a 5-point scale (1 = not at all, 5 = all the time). A sample item is “Why can’t I ever succeed?” Higher scores indicate more frequent negative automatic thoughts. The Turkish validity and reliability study of the ATQ was conducted by Aydın and Aydın (1990). Cronbach’s alpha in the present sample was 0.97.

General Self-Efficacy Scale: General self-efficacy was assessed using the 17-item General Self-Efficacy subscale of the Self-Efficacy Scale (Sherer et al., 1982), rated on a 5-point Likert scale (1 = does not describe me at all, 5 = describes me very well), with 11 items reverse-scored. A sample item is “When I make plans, I am certain I can make them work.” Higher scores indicate greater general self-efficacy. This instrument measures domain-general beliefs about one’s capacity for effective action and is explicitly distinct from measures of academic self-efficacy. The Turkish validity and reliability study of the scale was conducted by Yıldırım and İlhan (2010). Cronbach’s alpha was 0.86.

Frost Multidimensional Perfectionism Scale: Perfectionism was assessed using the 35-item Frost Multidimensional Perfectionism Scale (FMPS; Frost et al., 1990), comprising six subscales rated on a 5-point Likert scale (1 = strongly disagree, 5 = strongly agree). A sample item is “I should be upset if I make a mistake.” Higher total scores indicate greater perfectionism. The Turkish adaptation was developed by Kağan (2011), who confirmed the original six-factor structure in a Turkish university sample. Total scores were used in all analyses. Cronbach’s alpha was 0.91.

Locus-of-Control Scale: Locus of control was assessed using the 47-item scale developed by Dağ (2002) for Turkish samples, rated on a 5-point Likert scale (1 = not at all appropriate, 5 = completely appropriate), with 22 items reverse-scored. A sample item is “No matter what one does, nothing turns out the way one wants.” Higher scores indicate a more external locus of control. The scale is grounded in Rotter’s (1966) theoretical framework. Cronbach’s alpha was 0.87.

### 2.3. Data Analysis

Prior to analysis, data quality was evaluated using three sequential criteria targeting distinct sources of response invalidity. First, 17 participants who left more than 10% of the 252 items in the full academic-behavior survey battery unanswered (i.e., more than 25 items) were excluded due to excessive item-level missingness. Second, 49 participants who selected the same response option on 30 or more consecutive items were excluded as likely careless responders; this longstring index was computed on raw item responses prior to reverse-scoring. Third, 21 participants with multivariate outlier status at *p* < .001, identified via Mahalanobis distance (D^2^) computed across the total scores of the 12 scales in the full battery (the five focal scales and seven additional academic-behavior scales not analyzed here), were excluded (critical χ^2^(12) = 32.91). Screening was applied across the full battery rather than to the five focal scales alone because careless responding is a session-level response set rather than a scale-specific one. The three criteria flagged entirely non-overlapping sets of participants. In total, 87 participants were excluded across the three criteria, reducing the initial sample from 1090 to 1003. High scale scores were retained as theoretically meaningful; only statistically identified outliers were removed. A sensitivity analysis in which the 21 multivariate outliers were retained (N = 1024) yielded essentially unchanged model fit (CFI = 0.956, TLI = 0.943, RMSEA = 0.076, SRMR = 0.073), with no standardized path coefficient differing by more than 0.02 in absolute value. The non-significant direct effect of automatic thoughts on procrastination remained non-significant. The reported findings are therefore not attributable to these exclusions. 

Analyses proceeded in four sequential stages. First, descriptive statistics, reliability analyses, and group comparisons (independent-samples t tests and one-way ANOVAs) were computed. Second, the measurement model was evaluated using confirmatory factor analysis (CFA) with an item-parceling approach (Little et al., 2002). Parceling was adopted because the five instruments comprise 143 items in total, and modeling them at the item level would have produced an indicator-to-sample-size ratio unfavorable for stable estimation. Three parcels were formed for each construct by partitioning the items into consecutive blocks in their original scale order and averaging the items within each block. Because each scale was treated as unidimensional for the purposes of the structural model, parcels were not constructed to represent subscales; the trade-off is that parceling aggregates item-level information and therefore cannot detect misfit localized to individual items. Third, structural equation modeling was applied to test the proposed mediation model; indirect effects were evaluated using bootstrapping with 1000 resamples (Preacher & Hayes, 2008). Fourth, person-centered latent profile analysis (LPA; Spurk et al., 2020) was conducted to identify qualitatively distinct individual profiles within the sample, followed by a 10-fold cross-validated random forest analysis (Breiman, 2001) to assess the tenability of the linearity assumption underlying the structural model. Model fit was evaluated against established criteria: CFI ≥ 0.95, TLI ≥ 0.95, RMSEA ≤ 0.08, and SRMR ≤ 0.08 (Hu & Bentler, 1999). Analyses were conducted in lavaan using maximum likelihood estimation with robust (Huber–White) standard errors and scaled test statistics (MLR), given deviations from multivariate normality; robust fit indices are reported throughout. All analyses were conducted in R (version 4.6.0; R Core Team, 2026). Structural equation models were estimated with the lavaan package (version 0.6-21; Rosseel, 2012), latent profile models with the mclust package (version 6.1.2; Scrucca et al., 2023), and random forest analyses with the randomForest package (version 4.7-1.2; Liaw & Wiener, 2002).

## 3. Results

### 3.1. Descriptive Statistics and Correlations

Table 1 presents the descriptive statistics and the correlation matrix for the main variables. The skewness and kurtosis values of all variables were within acceptable bounds (|skewness| < 1). Academic procrastination showed a strong negative correlation with self-efficacy (r = −0.66), a moderate positive correlation with automatic thoughts (r = 0.42), and a low positive correlation with locus of control (r = 0.20). Notably, the direct correlation between perfectionism and procrastination was non-significant (r = 0.06), suggesting that the effect of perfectionism on procrastination may be only indirect.

### 3.2. Differences by Gender and Year of Study

Independent-samples t tests indicated no gender differences on most of the main variables. A significant difference emerged only for perfectionism, with men (M = 2.93) scoring higher than women (M = 2.80), t = −3.30, *p* = .001, d = 0.23 (Table 2). One-way ANOVA results revealed no significant effect of year of study on any main variable; the largest, for perfectionism, was non-significant and negligible in magnitude (F = 1.87, *p* = .134, η^2^ = 0.006). Age was negatively correlated with procrastination (r = −0.23, *p* < .001) and positively correlated with self-efficacy (r = 0.20, *p* < .001).

### 3.3. Measurement Model

The five-latent-factor measurement model was tested using an item-parceling approach (Little et al., 2002). The model showed an acceptable-to-good fit to the data: χ^2^(80) = 484.97, CFI = 0.958, TLI = 0.945, RMSEA = 0.074, SRMR = 0.069. TLI fell marginally below the conventional 0.95 threshold and RMSEA slightly exceeded the more stringent 0.06 criterion, although both remained within commonly applied bounds of acceptable fit. The standardized factor loadings ranged from 0.53 (one of the three locus-of-control parcels) to 0.95, with all remaining parcels loading above 0.76. Composite reliability (CR) values ranged from 0.79 to 0.95, and average variance extracted (AVE) values ranged from 0.56 to 0.87; all values exceeded the recommended thresholds (CR ≥ 0.70, AVE ≥ 0.50) (Table 3; Fornell & Larcker, 1981). Discriminant validity was supported by the Fornell–Larcker criterion: for every pair of constructs, the square root of the AVE exceeded the corresponding latent correlation. The narrowest margin involved self-efficacy and procrastination, whose latent correlation (−0.79) fell just below the square root of the AVE of self-efficacy (0.81) and more clearly below that of procrastination (0.86). The two constructs are thus empirically separable, although their close association warrants comment. They are distinguished conceptually as well as psychometrically: the self-efficacy items assess domain-general beliefs about one’s capacity for effective action, with no reference to academic tasks or to delay, whereas the procrastination items describe specific dilatory behaviors; the two scales also differ in response format and anchor wording. The confirmatory single-factor test reported below provides further evidence that the constructs are not reducible to a single underlying dimension.

Because all measures were self-reported within a single session, the possibility of common-method variance was examined using Harman’s single-factor test in its confirmatory form (Podsakoff et al., 2003). A model in which all 15 parcels loaded on a single latent factor fitted the data poorly, χ^2^(90) = 4956.41, CFI = 0.491, TLI = 0.406, RMSEA = 0.244, SRMR = 0.183, and was substantially inferior to the five-factor measurement model. Common-method variance is therefore unlikely to account for the observed pattern of associations, although this test cannot rule out its presence entirely.

### 3.4. Structural Model and Mediation

**Figure 1:**
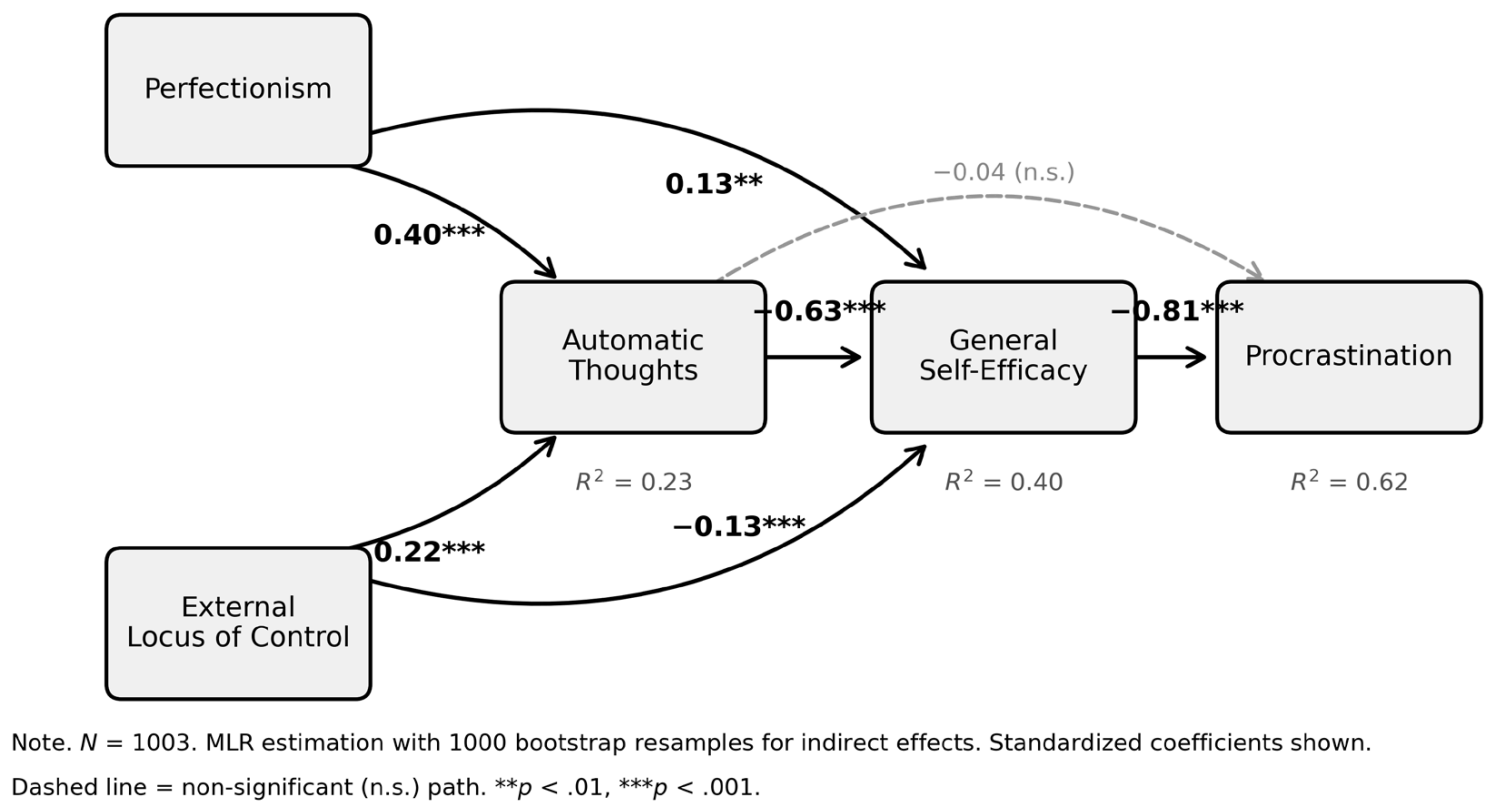
Structural Equation Model of Academic Procrastination (Standardized Coefficients).

The structural model showed an acceptable-to-good fit to the data: χ^2^(82) = 490.50, CFI = 0.958, TLI = 0.946, RMSEA = 0.074, SRMR = 0.070. The model accounted for 62% of the variance in procrastination, 40% in self-efficacy, and 23% in automatic thoughts. As shown in Figure 1, perfectionism (β = 0.40) and external locus of control (β = 0.22) positively predicted automatic thoughts. Automatic thoughts strongly negatively predicted self-efficacy (β = −0.63). Self-efficacy was additionally predicted, as direct paths, by perfectionism (β = 0.13, *p* = .001) and external locus of control (β = −0.13, *p* < .001); the positive perfectionism → self-efficacy direct path reflects a suppression effect that emerges once automatic thoughts are partialled out and produces the negative perfectionism → self-efficacy → procrastination indirect effect reported below. Most importantly, self-efficacy was the strongest predictor of procrastination (β = −0.81, *p* < .001), whereas the direct effect of automatic thoughts on procrastination remained non-significant (β = −0.04, *p* > .05). This pattern is consistent with a fully mediated structure in which automatic thoughts influence procrastination only through self-efficacy.

The bootstrap analysis revealed five significant indirect effects (Table 4). The strongest indirect path was the effect of automatic thoughts on procrastination through self-efficacy (indirect effect = 0.51, 95% CI [0.44, 0.58]). The effects of both perfectionism and locus of control on procrastination were mediated by self-efficacy. The indirect paths in which the upstream variables proceeded only through automatic thoughts while bypassing self-efficacy were non-significant, reinforcing the indispensable mediating role of self-efficacy.

### 3.5. Person-Centered Analysis: Latent Profile Analysis

**Figure 2:**
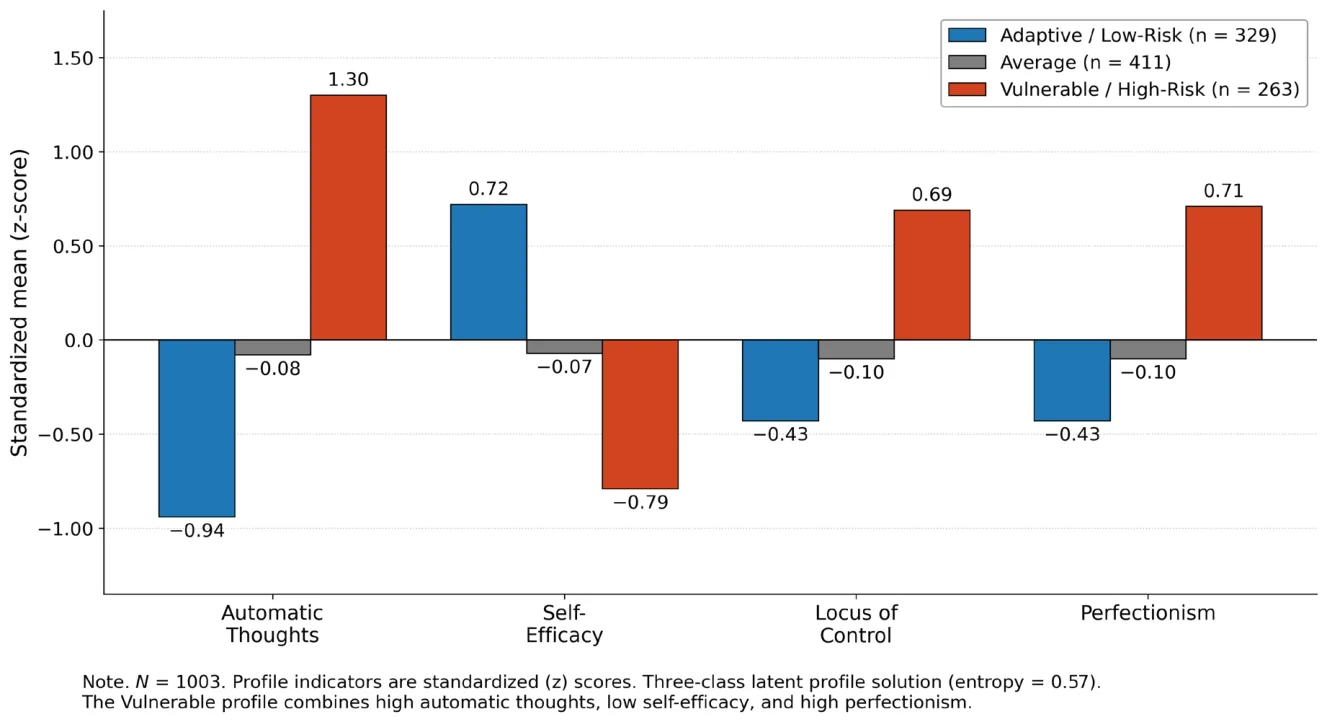
Standardized Indicator Means Across the Three Latent Profiles.

Latent profile models with one to four classes were estimated in mclust (version 6.1.2) using the four non-cognitive indicators (automatic thoughts, general self-efficacy, locus of control, and perfectionism); procrastination was excluded from the profile solution and retained as the outcome against which profiles were compared. Model comparison statistics are reported in Table 5. In mclust, BIC and related indices are parameterized such that higher values indicate better fit. On this criterion the three-class solution was optimal (BIC = −10,630.08), marginally ahead of the two-class solution (BIC = −10,631.59) and clearly ahead of the four-class solution (BIC = −10,678.07); the adjusted BIC likewise favored three classes. AIC continued to improve with additional classes, and the bootstrap likelihood-ratio test was significant at every step, favoring additional classes at each comparison (*p* = .01); neither index therefore provided a usable stopping rule in a sample of this size, a well-documented limitation in large samples (Nylund et al., 2007). The three-class solution was retained on the basis of BIC and adjusted BIC, class size, and substantive interpretability, since the four-class solution split the sample into a small additional class (*n* = 133) whose mean posterior classification probability was low (0.60). Because the two- and three-class solutions were nearly indistinguishable on BIC, and because classification certainty was only moderate (entropy = 0.571; mean posterior probabilities 0.75–0.86), the profiles are treated as provisional throughout. The three profiles shown in Figure 2 were labeled as follows: (a) an Adaptive/Low-Risk profile (*n* = 329; low automatic thoughts, high self-efficacy), (b) an Average profile (*n* = 411), and (c) a Vulnerable/High-Risk profile (*n* = 263; high automatic thoughts, low self-efficacy, high perfectionism). The profiles differed significantly in level of procrastination (η^2^ = 0.17). The vulnerable profile showed the highest level of procrastination (M = 2.66) and the adaptive profile the lowest (M = 2.04); the difference between the two extreme profiles was very large (Cohen’s d = 1.21).

### 3.6. Robustness Check: Random Forest

To assess the tenability of the linearity assumption, a random forest regression was compared with ordinary least squares regression using identical inputs: the four observed scale scores (automatic thoughts, general self-efficacy, locus of control, and perfectionism) as predictors and the observed procrastination score as the outcome. Both models were evaluated on the same 10-fold cross-validation partitions, with predictive accuracy indexed by the cross-validated coefficient of determination computed from out-of-fold predictions. The random forest was grown with 500 trees and the package default of one variable sampled at each split (mtry = p/3, rounded down). The two methods showed similar explanatory power (linear R^2^ = 0.44; random forest R^2^ = 0.42; ΔR^2^ = −0.02, *p* = .77), indicating no meaningful nonlinear pattern; because a random forest can also underperform ordinary least squares on genuinely linear data, this result is best read as an absence of detectable nonlinearity or higher-order interactions rather than as positive confirmation of linearity. Permutation-based variable importance, normalized to sum to one, identified general self-efficacy as the dominant predictor (0.63), followed by automatic thoughts (0.22), perfectionism (0.09), and locus of control (0.06), in agreement with the structural model. Because these analyses were conducted on observed scale scores rather than latent variables, the resulting R^2^ values are not directly comparable with the 62% of latent variance in procrastination accounted for by the structural model, which is corrected for measurement error.

## 4. Discussion

The present study examined academic procrastination within a framework of non-cognitive individual differences, testing whether general self-efficacy functions as a common pathway through which the effects of perfectionism, locus of control, and negative automatic thoughts converge on procrastinatory behavior. Three analytic approaches—structural equation modeling, latent profile analysis, and random forest regression—addressed complementary aspects of this model and are interpreted below in relation to the distinct questions each was intended to answer.

Before turning to interpretation, it is useful to state which of the study’s assumptions received support and which did not. H2 and H3 were supported: general self-efficacy predicted procrastination strongly and negatively, and the distal variables showed significant indirect effects on procrastination through the mediators specified in the model. H1 was supported only in part. Automatic thoughts and external locus of control predicted general self-efficacy in the expected negative direction, but perfectionism did not; its direct path to self-efficacy was positive once automatic thoughts were controlled, reflecting the suppression pattern discussed below. H4 was supported in the sense that distinguishable profiles emerged and differed substantially in procrastination, although the moderate classification certainty means this support is qualified. The linearity assumption underlying the structural model was not contradicted by the random forest comparison, and the assumption that the five constructs are empirically distinct was supported by the measurement model, if narrowly so for self-efficacy and procrastination. The interpretations that follow should be read against this pattern of qualified support.

### 4.1. Self-Efficacy as a Common Pathway

The most theoretically central finding is the dissociation between the direct and indirect effects of automatic thoughts on procrastination. The direct path was non-significant (β = −0.04). The indirect path through general self-efficacy, in contrast, was strong (indirect effect = 0.51). This pattern aligns with Bandura’s (1997) social cognitive theory, which positions self-efficacy as the proximal regulator of goal-directed behavior. Negative cognitions do not translate into behavioral avoidance directly. Instead, they erode the individual’s belief in their capacity for effective action, and it is this erosion that statistically accounts for delay. The present findings extend Klassen et al.’s (2008) work. The mediating role identified here belongs specifically to general self-efficacy, that is, stable beliefs about one’s overall capacity for effective action, rather than to academic self-efficacy tied to specific performance domains (cf. Bandura, 2006; G. Chen et al., 2001). This distinction matters theoretically. Procrastination appears to be associated not merely with doubts about competence in a particular subject. It reflects a more pervasive erosion of agentic belief about one’s general effectiveness.

Perfectionism showed a near-zero direct correlation with procrastination (r = 0.06). Yet its indirect effect through the automatic thoughts → self-efficacy → procrastination chain was significant. This apparent paradox helps clarify a persistent ambiguity in the literature. Van Eerde’s (2003) meta-analysis documented small and inconsistent direct relationships between perfectionism and procrastination across studies, a pattern that has sometimes been interpreted as evidence that perfectionism is unrelated to task delay. The present results suggest instead that the inconsistency reflects model misspecification: when the cognitive–motivational mediators through which perfectionism operates are omitted, its effects on procrastination are attenuated or masked by suppression. When those mediators are included, perfectionism’s influence becomes coherent: perfectionistic tendencies are associated with more frequent negative automatic thoughts about performance, which erode general self-efficacy, which in turn predicts procrastination. Substantively, the suppression pattern reported above indicates that perfectionism as a whole is not associated with lower self-efficacy; rather, its association with lower self-efficacy operates through the negative automatic thinking it accompanies. Once that cognitive pathway is accounted for, the residual variance in perfectionism shows a weak positive association with self-efficacy, plausibly reflecting the achievement-striving component of the construct, although the present total-score measurement cannot isolate this dimension. Sapancı (2021), using the same Turkish instrument, reported a substantial direct path from perfectionism to procrastination, whereas the present model yields a near-zero direct association. The difference is likely a measurement one: the earlier study modelled only perfectionistic concerns, while the present analysis uses the full scale. Aggregating facets appears to attenuate the direct path while leaving the indirect route intact. Ferrari’s (1992) conceptualization of perfectionism-driven procrastination as a form of self-protective avoidance is thus supported, but the present model specifies the cognitive–motivational mechanism more precisely.

The effects of external locus of control on procrastination operated entirely through indirect paths within the model, both through the automatic thoughts → self-efficacy chain and through a direct path to self-efficacy. This pattern is consistent with the theoretical account offered by Rotter (1966) and elaborated by Steel and König (2006): external locus of control weakens the perceived contingency between personal effort and outcomes, which in turn undermines beliefs about personal efficacy, reducing the motivational basis for task engagement. Locus of control was specified as influencing procrastination entirely through the mediators rather than through a direct path, consistent with its characterization as a distal antecedent rather than a proximal driver of task delay.

Together, these findings are consistent with a hierarchically ordered mediation model in which perfectionism and locus of control, as relatively stable, distal dispositional factors, are related to procrastination through automatic thinking and, in turn, general self-efficacy as the proximal mechanism. It should be emphasized that this ordering was specified on theoretical grounds and tested as a statistical mediation model; the cross-sectional data are compatible with, but do not establish, the causal sequence it describes. Self-efficacy thus appears to occupy the position through which the effects of diverse upstream non-cognitive individual differences converge on behavior, consistent with Bandura’s (1997) characterization of self-efficacy as the common currency of motivated behavior. The direction of this association is not settled in the literature: some studies model self-efficacy as the outcome of contextual support with procrastination as the mediating step (Duan et al., 2024), an ordering that the present cross-sectional design cannot rule out.

### 4.2. Person-Centered Findings

The latent profile analysis identified three distinct individual profiles that differed substantially in procrastination level, providing person-level evidence consistent with the variable-level mediation findings (Spurk et al., 2020). What the profile results add is that the risk factors co-occur: the vulnerable profile was defined by the simultaneous presence of high automatic thoughts, low general self-efficacy, and high perfectionism rather than by any one of them, and the gap separating it from the adaptive profile was large. This concentration of risk in a specific configural pattern has potential practical implications, provided the profile structure replicates: identifying students who simultaneously exhibit elevated perfectionism, frequent negative automatic thinking, and low general self-efficacy may allow for more precise targeting of intervention resources.

The person-centered results additionally reinforce the structural model’s core conclusion by identifying general self-efficacy, not automatic thoughts or perfectionism alone, as the factor whose level most consistently differentiates the adaptive from the vulnerable profile. Students in the adaptive profile had high self-efficacy despite moderate perfectionism in some cases, suggesting that self-efficacy beliefs serve a protective function that can buffer the effect of other individual-difference risk factors. This finding aligns with Bandura’s (1997) proposition that self-efficacy mediates the impact of environmental and dispositional adversity on motivated behavior. Because classification certainty was only moderate, however, this person-level convergence should be read as corroborating the variable-level pattern rather than as establishing discrete subgroups.

The cultural and institutional setting may bear on these results in ways that are worth specifying rather than merely noting. Turkish higher education is characterized by a highly competitive centralized placement examination, large course cohorts, and assessment concentrated in a small number of high-stakes examinations. Each of these features plausibly amplifies the pathway observed here: performance-contingent selection may heighten the salience of mistakes and thus the cognitive consequences of perfectionism, while infrequent assessment offers fewer of the incremental mastery experiences from which efficacy beliefs are built. In settings with continuous assessment, smaller cohorts, or less selective entry, the same constructs might be related differently, and the association between general efficacy beliefs and academic delay might be weaker where academic outcomes are less closely tied to a single evaluative event. Whether the ordering reported here is culturally general or specific to high-stakes educational systems is an empirical question that comparative designs could address.

### 4.3. Theoretical and Practical Implications

The present findings have implications for both theory and intervention design. Theoretically, they indicate that procrastination can be productively studied as a non-cognitive individual-differences outcome, one associated with a coherent configuration of dispositional, attributional, cognitive, and motivational characteristics that appear to be ordered through general self-efficacy. This positions the present study within the broader agenda of intelligence and individual-differences research that aims to characterize the non-cognitive determinants of academic functioning (Duckworth & Seligman, 2005; Zimmerman, 2000).

Three aspects of the present findings bear on existing theory more specifically. First, Temporal Motivation Theory treats expectancy as one of several inputs into a motivational calculus, alongside value, delay, and sensitivity to delay (Steel & König, 2006). The present pattern, in which a strong cognitive predictor of delay operated entirely through efficacy beliefs, suggests that expectancy may not sit alongside the other terms so much as absorb the influence of upstream dispositional variation, which would give it a structurally different status within the theory. Second, Bandura’s (1997) account holds that self-efficacy mediates the effect of dispositional and environmental adversity on action, but it is generally applied to task-specific beliefs; the finding that a domain-general measure carried the effect suggests that the mediating function does not require domain correspondence between belief and behavior, which is a stronger claim than the theory ordinarily makes. Third, the perfectionism result runs against the common assumption that perfectionism exerts a uniformly negative influence on self-regulation. Once its cognitive consequences were accounted for, the residual association with self-efficacy was positive, which is difficult to reconcile with accounts treating perfectionism as a unitary risk factor and more consistent with models distinguishing strivings from concerns (Stoeber & Otto, 2006).

For intervention, the findings raise the possibility that targeting automatic thoughts alone, without addressing the erosion of general self-efficacy beliefs that those thoughts produce, may be insufficient. Interventions that directly cultivate self-efficacy (e.g., through mastery experience structuring, verbal persuasion, or physiological-state reappraisal; Bandura, 1997) represent a plausible avenue to test, given the position self-efficacy occupies in the present model. Because no intervention was administered and no causal mechanism was manipulated here, however, the comparative effectiveness of self-efficacy-focused and cognition-focused approaches remains an open empirical question. At the same time, the person-centered results raise the possibility that the sub-group characterized by the full triad of elevated perfectionism, negative automatic thinking, and low general self-efficacy would benefit from multi-component interventions addressing all three risk factors rather than any one in isolation, although the provisional status of the profile solution means that such targeting would require replication before being translated into practice.

These implications address distinct audiences. For researchers in individual differences and educational psychology, the study offers a testable ordering of non-cognitive predictors and illustrates how variable-centered and person-centered evidence can be brought to bear on a single model. For university counseling and student support services, the results indicate that students presenting with frequent negative self-referential thinking and low general efficacy beliefs may warrant attention beyond time-management advice, which remains the most common institutional response to academic delay. For instructors and curriculum designers, the centrality of general rather than task-specific efficacy beliefs suggests that course structures affording repeated and attainable success experiences may matter more than exhortations to plan. For clinicians working within cognitive–behavioral frameworks, the findings locate procrastination within a familiar cognitive model and suggest that efficacy beliefs may be a useful additional target when delay persists despite cognitive restructuring.

### 4.4. Limitations and Future Directions

The study has several limitations that qualify the interpretation of its findings. First, the cross-sectional design precludes causal inference; the directionality of the proposed mediation pathways rests on theoretical grounds rather than on the data, which are equally consistent with statistically equivalent models (including a reverse ordering in which procrastination and low self-efficacy reinforce negative automatic thoughts), and therefore requires confirmation through longitudinal or experimental designs (Preacher & Hayes, 2008). Second, the sample was drawn from a single cultural context (Turkey), limiting the generalizability of the findings to other cultural settings; cross-cultural replication is warranted. Third, all data were obtained via self-report and collected in a single session, raising the possibility that common-method variance inflates observed correlations, particularly the strong self-efficacy–procrastination path. Although the confirmatory single-factor test argued against a dominant method factor, this test is a relatively weak diagnostic, and no further remedy (e.g., a marker-variable approach; Podsakoff et al., 2003) was applied; multi-informant or multi-method designs would provide a stronger test of the proposed pathways. Fourth, the moderate entropy of the LPA solution (0.57) indicates non-trivial classification uncertainty, so the three-class structure should be regarded as provisional pending replication. Fifth, perfectionism was modeled as a unidimensional total score, whereas contemporary models distinguish adaptive from maladaptive dimensions (Hewitt & Flett, 1991; Stoeber & Otto, 2006); future work should test whether the indirect pathway is carried specifically by the maladaptive, evaluative-concerns dimension. Taken together, these limitations bear directly on the strength of the claims that can be made. The cross-sectional design and reliance on self-report mean that the model is best read as a theoretically motivated account of covariation rather than an established causal sequence; the moderate entropy means that the profiles describe one defensible partition rather than firmly established subgroups; and the absence of an intervention component means that the applied recommendations offered above are hypotheses for future trials rather than evidence-based prescriptions.

These limitations point to four lines of work. The most direct is longitudinal: measuring the four constructs at intervals across a semester would establish whether changes in automatic thinking precede changes in efficacy beliefs and whether both precede changes in delay, which is the temporal claim the present design cannot test. Second, the model implies a manipulable target, and this is testable: an intervention that raises general self-efficacy without directly addressing negative cognition should reduce procrastination if the ordering proposed here is correct, whereas one that restructures cognition without shifting efficacy beliefs should not. Such a design would provide a stronger test of the pathway than any observational study and would simultaneously address whether the ordering is causal or merely covariational. Third, cross-cultural comparison is needed to determine whether the pattern reported here is specific to high-stakes, examination-centered educational systems or generalizes across institutional contexts. Fourth, multi-method assessment would address the common-method concern directly: behavioral indices of delay, such as time-stamped assignment submission or task-initiation latency in the laboratory, would allow the model to be tested without relying on a single self-report source, and informant reports of dispositional characteristics would similarly reduce shared method variance. Combining these approaches, for example an intervention study with behavioral outcome measures in more than one national context, would test the model’s core claims more stringently than any single design.

## 5. Conclusions

The present study indicates that academic procrastination is associated with an ordered configuration of non-cognitive individual differences, with general self-efficacy occupying the position of a common pathway through which the diverse effects of distal dispositional factors (perfectionism and locus of control) and proximal cognitive processes (negative automatic thoughts) converge on self-regulatory failure. The convergence of three methodologically distinct analytic approaches on this conclusion, and the identification of a high-risk profile characterized by the co-occurrence of elevated perfectionism, negative automatic thinking, and low general self-efficacy, make general self-efficacy a promising target for procrastination interventions. Whether strengthening self-efficacy reduces procrastination over time remains to be established through longitudinal and intervention research. More broadly, the study contributes to individual-differences research by specifying where a well-established motivational construct sits in relation to other non-cognitive predictors and to educational and clinical practice by identifying a point of intervention that is common to several distinct risk configurations.

## Figures and Tables

**Table 1:** Descriptive Statistics and Correlations for the Main Variables.

Variable	M	SD	Skew	1	2	3	4	5
1. Procrastination	2.35	0.57	0.04	—	0.42	−0.66	0.20	0.06
2. Automatic Thoughts	2.09	0.80	0.86		—	−0.56	0.32	0.39
3. Self-Efficacy	3.64	0.62	−0.43			—	−0.28	−0.15
4. Locus of Control	2.60	0.41	−0.41				—	0.19
5. Perfectionism	2.83	0.55	0.27					—

Note. N = 1003. Skew = skewness. *p* < .05 for r ≥ |0.07|; *p* < .001 for r ≥ |0.10|.

**Table 2:** Gender Comparisons on the Main Variables (Independent-Samples *t* Test).

Variable	Women M (SD)	Men M (SD)	t	*p*	d
Procrastination	2.34 (0.59)	2.38 (0.52)	−1.02	.308	0.07
Automatic Thoughts	2.08 (0.79)	2.11 (0.82)	−0.39	.697	0.03
Self-Efficacy	3.63 (0.63)	3.66 (0.59)	−0.68	.495	0.05
Locus of Control	2.60 (0.40)	2.58 (0.42)	0.87	.382	0.06
Perfectionism	2.80 (0.54)	2.93 (0.55)	−3.30	.001	0.23

Note. Women *n* = 717, men *n* = 283; gender data missing for 3 participants. d = Cohen’s d.

**Table 3:** Confirmatory Factor Analysis: Composite Reliability and Average Variance Extracted.

Factor	Cronbach’s α	CR	AVE
Procrastination	0.91	0.89	0.74
Automatic Thoughts	0.97	0.95	0.87
Self-Efficacy	0.86	0.84	0.65
Locus of Control	0.87	0.79	0.56
Perfectionism	0.91	0.88	0.71

Note. CR = composite reliability; AVE = average variance extracted.

**Table 4:** Standardized Indirect Effects (Bootstrap).

Indirect Path	Effect	95% CI LL	95% CI UL
Automatic Thoughts → Self-Efficacy → Procrastination	0.51	0.44	0.58
Perfectionism → Automatic Thoughts → Self-Efficacy → Procrastination	0.20	0.16	0.24
Locus of Control → Automatic Thoughts → Self-Efficacy → Procrastination	0.11	0.08	0.14
Locus of Control → Self-Efficacy → Procrastination	0.11	0.05	0.16
Perfectionism → Self-Efficacy → Procrastination	−0.10	−0.16	−0.04

Note. All indirect effects are significant (confidence intervals do not include zero). 1000 bootstrap resamples.

**Table 5:** Latent Profile Analysis: Model Comparison Statistics.

Classes	log L	Par.	BIC	AIC	aBIC	Entropy	BLRT *p*	Class Sizes	Mean Posterior
1	−5350.24	14	−10,797.22	−10,728.47	−10,752.76	—	—	1003	—
2	−5215.59	29	−10,631.59	−10,489.18	−10,539.48	0.617	.010	619/384	0.93/0.80
3	−5163.01	44	−10,630.08	−10,414.01	−10,490.34	0.571	.010	263/329/411	0.86/0.80/0.75
4	−5135.17	59	−10,678.07	−10,388.33	−10,490.68	0.605	.010	263/133/358/249	0.84/0.60/0.83/0.74

Note. N = 1003. Models estimated in mclust (VVV covariance structure) using four indicators; BIC, AIC, and aBIC are parameterized so that higher values indicate better fit. Par. = parameters; aBIC = sample-size-adjusted BIC; BLRT = bootstrap likelihood-ratio test versus the (k − 1)-class solution (100 replications); mean posterior = mean posterior classification probability.

## Data Availability

The data are available from the corresponding author upon reasonable request.
